# Matrix Rhythm Therapy (MRT) Along With Conventional Physiotherapy Proves to Be Beneficial in a Patient With Post-Operative Knee Stiffness in Case of Tibia-Fibula Fracture: A Case Report

**DOI:** 10.7759/cureus.45384

**Published:** 2023-09-16

**Authors:** Vaishnavi B Warutkar, Subrat Samal, Ruchika J Zade

**Affiliations:** 1 Department of Musculoskeletal Physiotherapy, Ravi Nair Physiotherapy College, Datta Meghe Institute of Higher Education and Research, Wardha, IND

**Keywords:** conventional physiotherapy, tibia fibula fracture, rehabilitation, physiotherapy, matrix rhythm therapy

## Abstract

Open fractures of the lower extremities are much more serious as compared to those of the upper extremities. Open fractures occur when the damaged bone is exposed to the external environment through injured soft tissue, increasing the risk of infection. The distal tibia can be fractured by a low-energy mechanism, such as rotational strain or perhaps a high-energy mechanism, such as motor vehicle accidents or falls from high altitudes. This case report is of a male individual who underwent an accident that led to a midshaft tibia and fibula fracture with lateral malleolus fracture. For that, he was operated on with open reduction and internal fixation (ORIF) with interlock nailing for a fracture of the tibia on the right side. A thorough physiotherapy protocol was set, which included matrix rhythm therapy (MRT), and improvements were seen in the outcome measures taken. The course of therapy improved the patient's state of well-being. Functional re-education increased the strength and endurance of the muscles. The patient also developed lower limb strength.

## Introduction

Open long bone fractures occur at an incidence rate of 11.5 per one lakh individuals [1]. Open fractures of the lower extremities are much more serious in comparison to those of the upper extremities [1]. Open fractures occur when the damaged bone is exposed to the external environment through injured soft tissue, increasing the risk of infection [2,3]. The most frequent open long-bone fracture is a tibial diaphyseal fracture [2]. The majority of open tibia/fibula fractures happen in a bimodal age group, with young men suffering from high-energy trauma and older women suffering from low-energy fragility fractures [1]. The distal tibia fracture occurs in 7% to 9% of lower limb fractures, with the fibula fractured in approximately 85% of those instances [4]. The distal tibia can be fractured by a low-energy mechanism, such as rotational strain or perhaps a high-energy mechanism, such as motor vehicle accidents or falls from high altitudes [4,5]. Due to their nature, origin, limited soft tissue coverage, and blood supply, these fractures commonly result in non-union and soft tissue sequelae [6,7].

The aim of tibial fixation surgery is to enhance fracture stabilization while limiting soft tissue damage from surgical repair [6]. Based on the situations, closed reduction and cast implementation, external fixation, an addition of restricted internal fixation with the application of external fixation, as well as ORIF with plates, nails, and screws could be utilized to manage distal tibia and fibula fractures [8,9]. The surgeon faces a severe task in managing these fractures. Traditional surgical procedures involve ORIF as well as restricted internal fixation with external fixation. The capacity to anatomically decrease displaced fractures, especially articular fractures, is generally favored by open reduction and internal fixation. ORIF, on the other hand, usually necessitates two different incisions: one on the medial side to reach the distal tibia and one on the lateral side to reach the distal fibula [10].

Matrix rhythm therapy (MRT) is a technique employed in health centers today to cure abnormalities since it preserves the body's various physiological functions, adopting a cell-based, goal-oriented approach occurring at the cellular level [11]. The development of MRT is an external and dynamic method that permits cellular movement of tissues and activates matrix fluid using vibrations at an intensity of 8-12 Hz [11].

This case study refers to an individual who sustained injuries in an accident that caused a midshaft tibia and fibula fracture as well as a lateral malleolus fracture. He had undergone ORIF with interlock nailing for the fracture of the right tibia. A post-op, thorough rehabilitation program was necessary to minimize the repercussions of the operations and improve his quality of life. His physiotherapy protocol included matrix rhythm therapy (MRT) along with conventional physical therapy sessions.

## Case presentation

A male patient aged 35 years was brought to casualty with a history of road traffic accidents due to a slip and fall from a two-wheeler. Immediately after the trauma, he developed pain that was sharp shooting in character, sudden in onset, and gradually progressive over his right leg. The pain was aggravated by movement and relieved by rest. The pain was associated with the development of diffuse swelling over the right lower limb. No history of head injury, ENT bleeding, or loss of consciousness. A history of alcohol consumption was present.

The patient was taken to a local hospital, where he was managed with slab application and medication, and then he came to AVBRH (Acharya Vinoba Bhave Rural Hospital) for definitive management.

Clinical findings

Before the examination, proper consent from the patient was obtained. The patient was evaluated in the supine lying position. On observation of the right knee, the overlying skin appears to be normal. Diffuse swelling was present over the right lower limb. On palpation, the local temperature was raised. Bony tenderness was present over the midshaft tibia, fibula, and lateral malleolus. The knee range of motion (ROM) was restricted and painful. The ankle ROM was full, with active toe movements present.

Timeline

Table 1 shows the timeline for the patient.

**Table 1:** Timeline

Date of fall	14-09-22
Date of admission in the hospital	14-09-22
Date of surgery	19-09-22
Date of physiotherapy reference	23-09-22

Diagnostic assessment

Tables 2, 3 show the assessment of the patient’s pre-rehabilitation.

**Table 2:** Range of motion assessment preoperative NA: Not assessed.

Right joint	Active	Passive	Left joint	Active	Passive
Knee flexion	NA	NA	Knee flexion	0-125^0^	0-130^0^
Extension	NA	NA	Extension	125^0^-0	130^0^-0
Ankle Plantarflexion	NA	NA	Ankle plantarflexion	0-35^0^	0-45^0^
Dorsiflexion	NA	NA	Dorsiflexion	0-10^0^	0-15^0^
Inversion	NA	NA	Inversion	0-25^0^	0-30^0^
Eversion	NA	NA	Eversion	0-10^0^	0-15^0^

**Table 3:** Manual muscle testing assessment pre-operative

		Right	Left
Knee	Flexors	NA	4/5
	Extensors	NA	4/5
Ankle	Plantar flexors	NA	4/5
	Dorsi flexors	NA	4/5

Pain assessment

On NPRS, it was 6/10 at rest, and on movement, it was 7/10.

Medical management

Under spinal and epidural anesthesia, cleaning, painting, and draping of the right lower limb were done. A 5cm incision was made in the patellar tendon, both vertically and midline. Entry was made with tibia bone, guide wire was passed, and reduction was done with traction and manipulation. Proximal locking was done using two screws in the mediolateral plane. Distal locking was done with one cortical screw in the mediolateral plane and one screw in the anteroposterior plane. The reduction and implant were confirmed under the C-arm, and the reduction was found to be satisfactory. Then sterile dressing was done; and an above-knee slab was given. In this way, ORIF with interlock nailing for a fracture of the tibia's right side was done.

Physiotherapy protocol

A customized physiotherapy plan was set for the patient. He had physical therapy rehabilitation for 10 weeks, six days/per week.

Phase 1: Week 1

For 10 minutes, ice packs were used to decrease the swelling and redness. A knee brace was used the entire time. Ten repetitions of active-assisted right hip and knee ROM exercises performed twice daily. Complete range of motion exercises with weight cuffs for the left lower extremity and both upper extremities. For the quadriceps, hamstrings, and glutei muscles, static contraction workouts with 10 repetitions and a 10-second hold each were carried out. Every workout was done twice each day. Gait training was started by supporting the right lower extremity with a long knee brace and non-weight bearing (NWB) while using a walker.

Phase 2: Weeks 2-6

To prevent contracture in the flexion, the knee was kept extended. Exercises from the Phase 1 plan were continued. Inflammation was managed by continuing to use cryotherapy. Exercises to increase core stability were done for 10 repetitions with 5-second holds. With an increase in repetitions and intensity, non-affected upper and lower limbs were strengthened. The right lower limb's strength training was advanced to an active, pain-free range of motion. NWB was continued and advanced in order to perform functional activities independently under the required supervision. Every activity was done twice each day.

Phase 3: Weeks 6-8

The same routines from Phase 2 were continued. Right knee ROM was gradually started in a supine position with active assistance within a pain-free range. Dynamic quadriceps training was advanced with minimal to no support. Weight-bearing was started during gait training, with 25% weight bearing, and that much progressed every two weeks. In parallel bar setups, gait training was conducted to adjust to the weight-bearing position. Matrix rhythm therapy (MRT) was started and applied for 25 mins, five days/week.

Phase 4: Weeks 8-10

Exercises from all the above phases were advanced. An increase in repetitions and hold times started to advance the right-side lower extremity muscles' resistance training. After achieving good strength in the quadriceps and hamstring muscles, the use of the knee brace was discontinued. Gait training that included weight bearing advanced by 50%, which boosted the patient's self-assurance in their ability to walk on their own. The patient was told to begin 100% weight bearing only after the orthopedic surgeon had given instructions on radiographic confirmation of fused fracture fragments and a follow-up physical therapy session to teach the essential improvements. MRT was continued for 30 mins, five days/week.

The rehabilitation program according to phases is given below in Table 4.

**Table 4:** Phase-wise rehabilitation protocol.

Phase 1 (Week 1)	Phase 2 (Week 2-6)	Phase 3 (Weeks 6-8)	Phase 4 (Week 8-10)
Ice pack to decrease redness and swelling for 10 minutes.	Cryotherapy was continued to control inflammation.	The same phase-2 exercises were continued.	Phase 3 exercises progressed.
Active-assisted exercise to the right hip and knee and resisted exercise to the left lower extremity and bilateral upper extremity exercises with weight cuffs 10 repetitions, two sets.	To prevent contracture in the flexion, the knee was kept extended. With an increase in repetitions and intensity, non-affected upper and lower extremities were strengthened.	In the supine lying position, right knee ROM slowly started to active-assisted within a pain-free range.	After achieving good strength in the quads and hams, the use of the knee brace was discontinued.
For the quadriceps, hamstrings, and glutei muscles, static contraction workouts with 10 repetitions and a 10-second hold each were carried out. Every workout was done twice each day.	Core stability exercises were performed. The right lower limb's strength training was advanced to an active, pain-free range of motion. Dynamic quadriceps exercises were started in a pain-free range.	Dynamic quadriceps training was advanced with minimal to no support.	Advanced strengthening was started along with weight transfers. An increase in repetitions and hold times were used to develop the right-side lower limb muscles' resistance training.
Gait training was started by supporting the right lower extremity with a long knee brace and non-weight bearing (NWB) while using a walker.	Non-weight bearing (NWB) continued and advanced to perform functional activities independently under the required supervision. Every activity was done twice each day.	Weight-bearing was started during gait training, with 25% weight bearing, and that much progressed every two weeks. In parallel bar setups, gait training was conducted to adjust to the weight-bearing position.	Gait training that included weight bearing advanced by 50%, which boosted the patient's self-assurance in their ability to walk on their own. The patient was told to begin 100% weight bearing only after the orthopedic surgeon had given instructions on radiographic confirmation of fused fracture fragments and a follow-up physical therapy session to teach the essential improvements.
MRT was started in Phase 3.	MRT was started in Phase 3.	MRT was given for 25 mins, five days/week.	MRT was given for 30 mins, five days/week.

Follow-up and outcome

After receiving physical therapy for 10 weeks, the patient showed significant progress. Tables 5, 6 show the assessment of the patient post-rehabilitation.

**Table 5:** Range of motion assessment of the right lower limb post-rehabilitation

Joint	Active	Passive
Knee flexion	0-125^0^	0-130^0^
Ankle plantarflexion	0-35^0^	0-45^0^
Dorsiflexion	0-10^0^	0-15^0^
Inversion	0-25^0^	0-30^0^
Eversion	0-10^0^	0-15^0^

**Table 6:** Manual muscle testing assessment of the right lower limb post-rehabilitation

Joint	Muscle	Post-rehabilitation
Knee	Flexors	4/5
	Extensors	4/5
Ankle	Plantar flexors	4/5
	Dorsi flexors	4/5

Pain assessment

Pre-rehabilitation: NPRS 6/10 at rest, 7/10 on movement. Post-rehabilitation: NPRS 1/10 at rest, 3/10 on activity. Table 7 shows the phase-wise scoring of the LEFS (lower extremity functional scale).

**Table 7:** Score of the lower extremity functional scale (LEFS)

Week 1	Week 2-6	Week 6-8	Week 8-10
06	19	41	70

LEFS: It is a scale that contains 20 items, showing a score about a person’s ability to perform everyday tasks. The maximum score is 80. The lower the score, the greater the disability.

## Discussion

Fibular fractures are frequent, especially those that involve the ankle and the shaft just proximally. They generally follow mild traumas. Primary care and emergency clinicians frequently give the first management; therefore, they should be aware of these injuries. There have been many different approaches for treating this condition, from non-surgical approaches like plaster immobilization to surgical ones like ER, IM nailing, or plate fixation. In addition to the stress brought on by the need for surgery, hospital stays, and rehab programs, the consequences can also be very difficult for patients [12]. After a fracture, there is a substantial risk of non-union. Refracture, deformity, leg-length disparity, stiffness, discomfort, and dysfunction might exacerbate any union that is made [13].

More wound complications were seen in individuals lacking fibula fixation [14]. A tibial malalignment occurred in two individuals who had ORIF and six patients who underwent intramedullary nailing. Moreover, we discovered no distinction between ORIF and IM nailing in terms of the time to union, non-union, hardware failure, or deep abscesses. Our findings imply that IM nailing of distal tibial fractures presents challenges for management. An open diaphyseal tibial fracture that can be closed more cost-effectively using IM nailing than EF (external fixation). Furthermore, IM nailing had higher union rates after three months than EF [15]. MIPO (minimally invasive plate osteosynthesis) is a successful therapy for closed, unstable distal tibia fractures, eliminating the disadvantages related to more conventional techniques of internal fixation and/or ER [16].

A study done by Shrivastava S. says that MRT helps to reduce swelling and pain, improve joint motility, increase tissue elasticity, and aid in the restoration of functions [17]. Even the research done by Taspinar et al. says that MRT, along with massage, increases peripheral blood flow [18]. Research performed by Narin et al. suggests that when MRT is used alone, it is unable to decrease lymphedema; however, if continued therapy is given, it may have beneficial consequences [19].

## Conclusions

The course of therapy improved the patient's state of well-being. Through functional re-education, the muscles gained more power and endurance. Matrix rhythm therapy, along with conventional physical therapy, helps to increase the range of motion of the joint, enhance skeletal muscle repair, and promote regeneration of the injured muscles, thereby fastening the recovery by increasing the strength and resilience of the muscles of the lower limbs, which will ultimately result in improved quality of life.
